# ﻿Taxonomic notes of subgenus *Velia* (*Cesavelia*) Koçak & Kemal, 2010 (Hemiptera, Heteroptera, Veliidae) from China, with description of one new species

**DOI:** 10.3897/zookeys.1149.96680

**Published:** 2023-02-22

**Authors:** Zezhong Jin, Siying Fu, Zhen Ye

**Affiliations:** 1 Institute of Entomology, College of Life Sciences, Nankai University, Tianjin, 300071, China Nankai university Tianjin China

**Keywords:** Distribution, morphology, new record, range extension, semiaquatic bugs, taxonomy

## Abstract

Velia (Cesavelia) bui**sp. nov.** from Hubei Province, China is described, and Velia (Cesavelia) tonkina Polhemus & Polhemus, 2003 is newly recorded from China. In addition, new distribution data for three species of Velia (Cesavelia), *V.longiconnexiva* Tran, Zettel & Buzzetti, 2009, *V.sinensis* Andersen, 1981 and *V.tonkina* Polhemus & Polhemus, 2003 are provided. Photographs of the habitus in dorsal and lateral views, metafemora of males, genitalic structures and habitats, along with a distribution map of this subgenus, are provided.

## ﻿Introduction

The genus *Velia* Latreille, 1804, includes three subgenera: V. (Velia) Latreille, 1804, V. (Plesiovelia) Tamanini, 1955 and V. (Cesavelia) Koçak & Kemal, 2010. The subgenus Velia s.str. is monotypic and only includes one extant species, V. (Velia) rivulorum (Fabricius, 1775), which is distributed in the western Mediterranean (Tamanini 1947; Andersen 1995; Berchi et al. 2018). The subgenus Plesiovelia contains 28 taxa (23 species and 5 subspecies), distributed from western Europe to northwestern India, with extension to northern Africa (Andersen 1981, 1995b; Tran et al. 2009; Berchi et al. 2018). The subgenus Cesavelia is restricted to the Oriental Region, i.e., northern India, Nepal, central and southern China and northern Vietnam (Andersen 1981; Tran et al. 2009; Koçak and Kemal 2010; Basu et al. 2013). This subgenus was originally named *Haldwania* Tamanini, 1955 and this name was used in subsequent studies, e.g., Andersen (1981), Polhemus and Polhemus (1998), and Tran et al. (2009). It was then replaced by the present subgeneric name, because it was determined to be a junior homonym of *Haldwania* Beier, 1930 (Mantodea) (Koçak and Kemal 2010).

Morphologically, *Cesavelia* can be distinguished from the other subgenera by the relatively long antennal segment I (i.e., longer than width of head across eyes) and less stout hind femur (Tamanini 1955a, b, c; Andersen 1981; Berchi et al. 2018). Hitherto, ten species have been considered valid in this subgenus (Tamanini 1955b; Andersen 1981; Polhemus and Polhemus 1998; Tran et al. 2009; Basu et al. 2013), but only three species have been recorded from China before this study: *V.longiconnexiva* Tran, Zettel & Buzzetti, 2009, *V.sinensis* Andersen, 1981, and *V.yunnana* Tran, Zettel & Buzzetti, 2009. Here we report a new species Velia (Cesavelia) bui sp. nov. from Hubei Province of China, which extends the known distribution range of this subgenus eastward into central China. In addition, *V.tonkina* Polhemus & Polhemus, 2003 is recorded from China for the first time, and new distribution data for three species, *V.longiconnexiva*, *V.sinensis* and *V.tonkina* are provided. This paper also provides photographs of the habitus in dorsal and lateral view, metafemora of males, genitalic structures, habitats of species occurring in China, and a distribution map of this subgenus.

## ﻿Material and methods

All the specimens examined in this study are deposited in the Institute of Entomology, College of Life Sciences, Nankai University, Tianjin, China (**NKUM**). All measurements are given in millimeters (mm). The illustrations of specimens in dorsal view and structural details were captured using a Nikon SMZ1000 stereomicroscope equipped with a computer-controlled SPOTRT digital camera and Helicon software (Helicon Remote ver. 3.9.12 W and Helicon Focus ver. 7.7.5). The skeletal elements of genital segments were dissected after macerated with 5% KOH. The photographs of the dissected male genital segments were made using an OLYMPUS BX53 microscope equipped with a computer-controlled Canon OLYMPUS DP72 digital camera and cellSens Standard ver. 1.6 software.

## ﻿Taxonomic accounts

### ﻿Family Veliidae Brullé, 1836

**Subfamily Veliinae Brullé, 1836**

#### ﻿Genus *Velia* Latreille, 1804

##### ﻿ Velia (Cesavelia) bui sp. nov.

Taxon classificationAnimaliaHemipteraVeliidae

E4608581-68B7-5BB2-B6C0-E620FF1C86D1

https://zoobank.org/40E8683C-27BF-43A4-A29C-ACE62F1BA7C5

**Figure 1.**
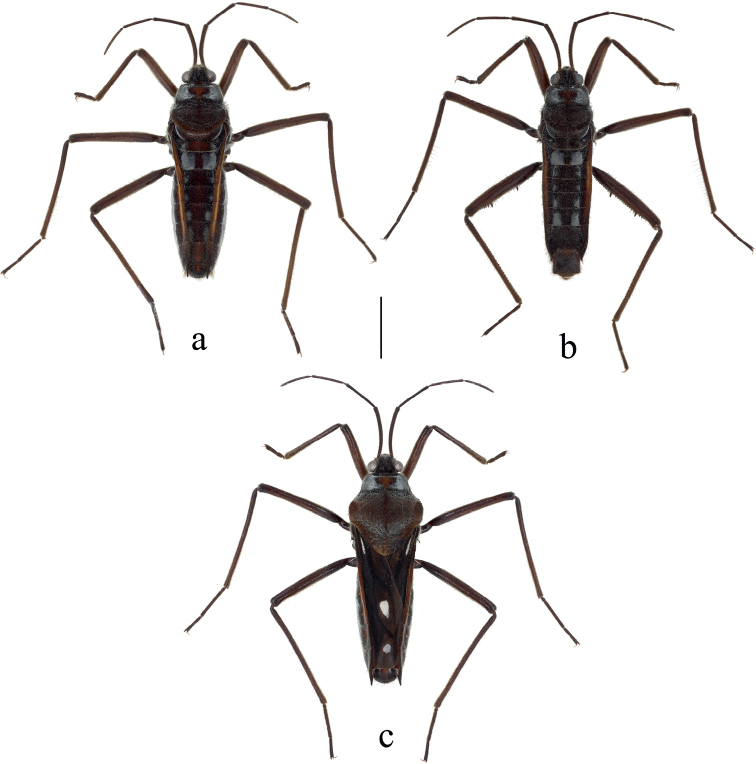
Habitus of females and male of Velia (Cesavelia) bui sp. nov. in dorsal view **a** apterous female **b** apterous male **c** macropterous female. Scale bar: 2.0 mm.

Figs 1a–c , 3a, b , 5a , 6a, b , 7a, e , 8a–c , 9a, b

###### Material examined.

***Holotype***: apterous ♂, China, Hubei Province, Wufeng County, Houhe National Nature Reserve: 30.0869°N, 110.5520°E; 1085 m a.s.l.; 2015-VIII-8; Zhen Ye leg. (NKUM). ***Paratypes***: 1 apterous ♀ 1 macropterous ♀, same data as holotype (NKUM).

###### Diagnosis.

Body large, mainly brown. Connexiva straight in dorsal view, with dark yellow strips in male and brighter strips in female (Figs 1a–c, 3a, b), connexival spines sharp and dorsocaudally directed in female (Fig. 3a, b); abdominal segment VIII of male stout and ventrally concaved (Fig. 6a, b); proctiger of male with triangular dilations on each side and broadly rounded hind margin (Fig. 7a).

###### Comparative notes.

*Veliabui* sp. nov. and *V.longiconnexiva* are similar in the coloration and size of the body. However, the female of *V.bui* sp. nov. can be easily distinguished from that of *V.longiconnexiva* by its nearly straight connexiva and relatively slender, straight, sharp, slightly directed dorsad connexival spines (Fig. 3a, b vs. 3c, d). The male of the new species can be distinguished from that of *V.longiconnexiva* by its relatively stout segment VIII in lateral view and slightly emarginated dorsal hind margin (Fig. 6a, b vs. 6c, d), the triangular lateral dilations and the broadly rounded hind margin of proctiger (Fig. 7a vs. 7b).

###### Description of apterous male

**(holotype). Measurements.** Body: length 7.00, width 1.90. Head: length 0.58, width: 1.13, width about 1.95 times length. Antenna: 4.97 (1.63+1.13+1.13+1.08), length of antennal segment I about 1.44 times head width. Pronotum: width about 1.03 times length (length 1.48, width 1.53). Lengths of leg segments (femur: tibia: tarsus (tarsal segment I + segment II + segment III)): fore leg: 2.13: 2.13: 0.73 (0.05+0.25+0.43); middle leg: 3.13: 3.30: 1.88 (0.13+1.00+0.75), length of mesotarsus II about 1.33 times length of mesotarsus III; hind leg: 2.95: 3.38: 1.66 (0.08+0.95+0.63), length of metatarsus II about 1.51 times length of metatarsus III. Abdominal segment VIII: length about 1.67 times width (length 1.64, width 0.98).

**Color** (Fig. 1b). Body mainly brown, with scattered silvery pubescence. Pronotum with a row of black punctures near anterior margin and other punctures scattered on posterior lobe. Median part of anterior pronotal lobe and midline of pronotum dark orange; metanotum completely dark brown. Sides of abdomen dark brown, with dark orange stripes along connexiva. Silvery pubescence usually distinctly denser on anterolateral corners of pronotum, lateral corners of metanotum and lateral parts of abdominal mediotergites.

**Structure.** Body relatively large, covered with dense, short pubescence. ***Head*** (Fig. 1b): triangular, almost perpendicular to thorax, without deflection; anteclypeus and postclypeus with dense, peg-like setae; antennal sockets prominent, antennal segment I much longer than head width, slightly thicker than antennal segments II–IV. ***Thorax*** (Fig. 1b): pronotum slightly wider than length, hind margin of pronotum broadly rounded, lateral parts of pronotum medially with distinct constrictions, middle part slightly raised and lateral parts of anterior pronotal lobe concaved; mesonotum completely hidden beneath pronotal lobe and hind part of metanotum visible in dorsal view; lateral evaporatoriums slender, with a cluster of suberect, thick setae on each side; legs mainly with decumbent or suberect setae, tarsi of fore legs short, tarsi of middle and hind legs long and slender; profemora moderately incrassate, slightly curved and contracted subapically; mesofemora medially slender, mesotibiae slender and ventrally with a row of long, erect setae on each side; metafemora (Fig. 5a) relatively stout, ventrally with two rows of small teeth and two prominent long teeth on each side, metatibiae ventrally with two rows of small spines and dorsally with a row of suberect setae on each side. ***Abdomen*** (Figs 1b, 6a, b): relatively slender; mediotergite I concave laterally, mediotergites II–VII almost flat; connexiva moderately raised, almost parallel without convergence, connexival spines sharp, caudally pointed; abdominal segment VIII (Fig. 6a, b) relatively stout, ventrally concaved in lateral view, posteriorly with dense setae, dorsal hind margin of abdominal segment VIII medially emarginated. ***Genital segments*** (Figs 7a, 8a–c, 9a, b): relatively large and visible in vitro; proctiger (Fig. 7a) relatively flat, with a triangular dilation on each side, posteriorly with short, sparse setae; paramere (Fig. 8a–c) sickle-shaped, relatively slender, with thick setae on external side, apexes slightly blunt, subapical part with distinct dilation; endosoma (Fig. 9a, b) stout, apical ends of lateral sclerites distinctly constricted, dorsal sclerites weakly sclerotized, translucent and curved, secondary ventral sclerite slender, accessory sclerite absent.

###### Description of apterous female.

**Measurements.** Body: length 7.30, width: 2.13. Head: length 0.80, width: 1.13, width about 1.41 times length. Antenna: 5.02 (1.63+1.13+1.13+1.13), length of antennal segment I about 1.44 times head width. Pronotum: width about 1.08 times length (length 1.70, width 1.83). Length of leg segments (femur: tibia: tarsus (tarsal segment I + segment II + segment III)): fore leg: 2.13: 2.13: 0.76 (0.08+0.25+0.43); middle leg: 3.13: 3.25: 1.89 (0.13+1.13+0.63), length of mesotarsus II about 1.79 times length of mesotarsus III; hind leg: 3.00: 3.38: 1.59 (0.08+0.88+0.63), length of metatarsus II about 1.40 times length of metatarsus III.

**Color** (Figs 1a, 3a, b). Similar to apterous male with following exceptions: hind margin of pronotum, median part of metanotum and all mediotergites dark orange. Stripes along connexiva much brighter.

**Structure.** Body slightly larger than apterous male. ***Head*** (Figs 1a, 3a, b): Similar to apterous male with following exceptions: the antennal segment I more bent. ***Thorax*** (Figs 1a, 3a, b): similar to apterous male with following exceptions: posterior pronotal lobe distinctly wider than anterior pronotal lobe; profemora much slender; metafemora slender, ventrally with two rows of small spines on each side, metatibiae ventrally without any spines or teeth. ***Abdomen*** (Figs 1a, 3a, b): similar to apterous male with following exceptions: relatively stout; connexiva gradually convergent toward abdominal apex, connexival spines long, slender and straight, slightly dorsocaudally directed. ***Genital segments***: gonocoxae and gonapophyses semi-membranous, rami strongly sclerotized; proctiger (Fig. 7e) broad, sub-circle, posteriorly with short, sparse setae.

###### Description of macropterous female.

**Measurements.** Body: length 7.50, width 2.38. Head: length 0.72, width 1.18, width about 1.64 times length. Antenna: 5.12 (1.68+1.13+1.18+1.13), length of antennal segment I about 1.42 times head width. Pronotum: width about 0.90 times length (length 2.63, width 2.38). Lengths of leg segments (femur: tibia: tarsus (tarsal segment I + segment II + segment III)): fore leg: 2.13: 2.13: 0.71 (0.08+0.25+0.38); middle leg: 3.00: 3.38: 1.82 (0.05+1.04+0.73), length of mesotarsus II about 1.42 times length of mesotarsus III; hind leg: 3.00: 3.35: 1.64 (0.08+0.93+0.63), length of metatarsus II about 1.48 times length of metatarsus III. Wing: length: 4.65, width: 1.16.

**Color** (Fig. 1c). Similar to apterous female with following exceptions: hind margin of pronotum medially orangish; forewing brownish with dark brown veins and three white spots; sides of abdominal segments III–VI including connexiva with dark orange marks.

**Structure.** Body slightly larger than apterous female. ***Head*** (Fig. 1c): similar to apterous female. ***Thorax*** (Fig. 1c): similar to apterous female with following exceptions: pronotum large, nearly pentagonal, with broad posterior lobe completely covering the meso- and metanotum, humeral corners prominent; each forewing with three spots (Fig. 1c): a thin spot in first basal cell, a large teardrop-shaped spot in apical cell and a suborbicular spot between the free apical veins. ***Abdomen and genital segments***: similar to apterous female.

###### Macropterous male.

Unknown.

###### Etymology.

The species is named in honor of Prof. Wenjun Bu (NKUM) for his outstanding contribution to the studies on Chinese fauna of Heteroptera, on the occasion of his 60^th^ birthday.

###### Distribution.

China (Hubei) (Fig. 11).

##### ﻿ Velia longiconnexiva

Taxon classificationAnimaliaHemipteraVeliidae

Tran, Zettel & Buzzetti, 2009

5A741975-8991-5B5A-83B6-0456EAC3AAFC

**Figure 2.**
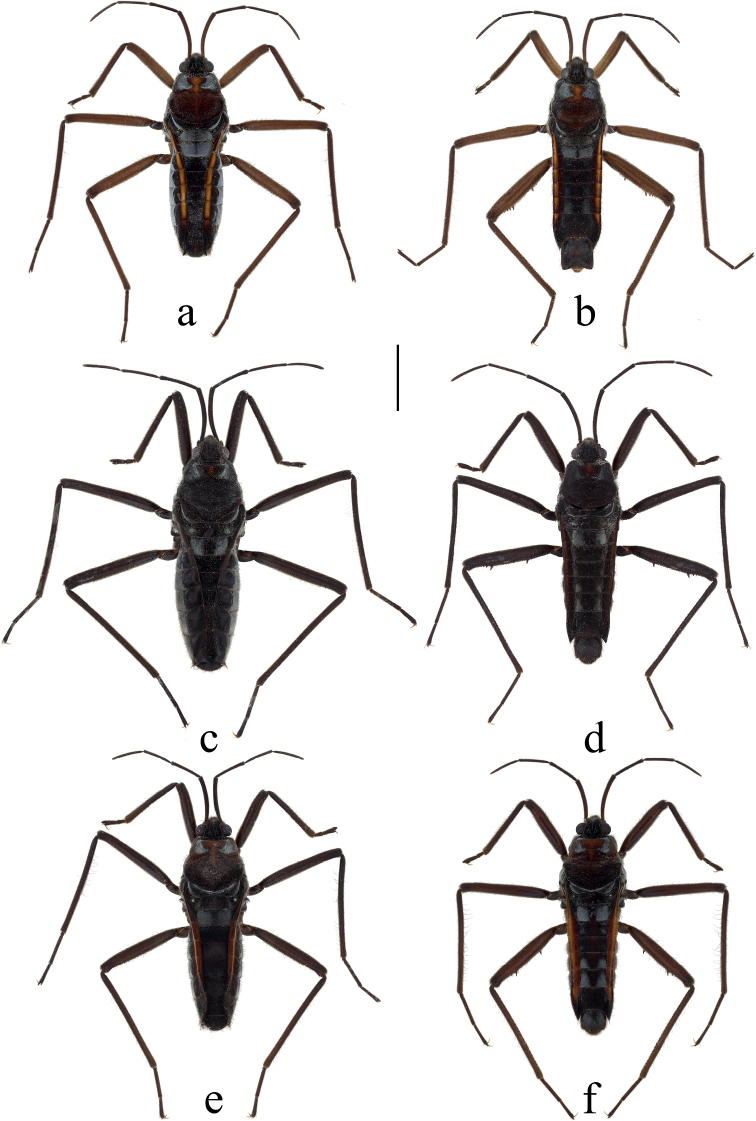
Habitus of females and males of *Velia* spp. in dorsal view (apterous form) **a***V.longiconnexiva*, female **b***V.longiconnexiva*, male **c***V.sinensis*, female **d***V.sinensis*, male **e***V.tonkina*, female **f***V.tonkina*, male. Scale bar: 2.0 mm.

**Figure 3.**
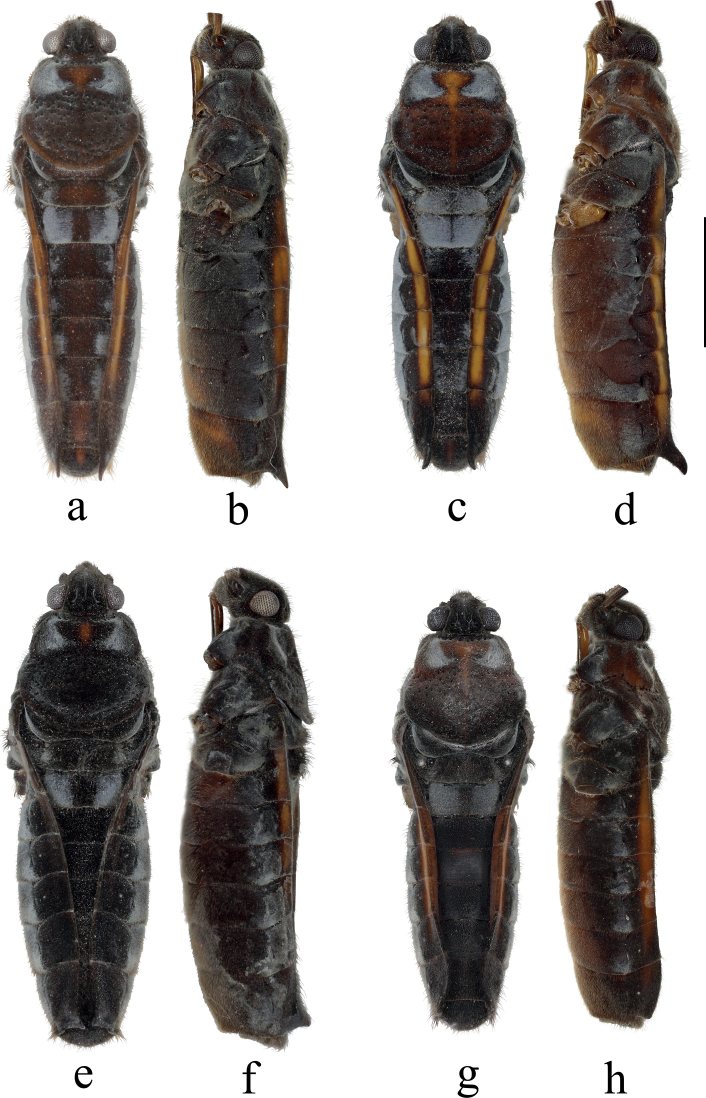
Bodies of *Velia* spp. (apterous female) **a***V.bui* sp. nov., dorsal view **b***V.bui* sp. nov., lateral view **c***V.longiconnexiva*, dorsal view **d***V.longiconnexiva*, lateral view **e***V.sinensis*, dorsal view **f***V.sinensis*, lateral view **g***V.tonkina*, dorsal view **h***V.tonkina*, lateral view. Scale bar: 2.0 mm.

Figs 2a, b , 3c, d , 5b , 6c, d , 7b, f , 8d–f , 9c, d

###### Material examined.

8 apterous ♂♂ 9 apterous ♀♀, China, Guizhou Province, Leishan County, Leigongshan National Nature Reserve: 26.3827°N, 108.2277°E; 1700 m a.s.l.; 2013-VIII-3; Zhen Ye leg. (NKUM).

###### Diagnosis.

Body large, mainly dark brown. Connexiva with dark yellow strips in male and brighter strips in female (Figs 2a, b, 3c, d), connexival spines of female long, dorsocaudally directed (Fig. 3c, d); abdominal segment VIII of male slender, dorsal hind margin strongly emarginated, ventrally concaved in lateral view (Fig. 6c, d); proctiger of male with sub-trapezoid dilations on each side and emarginated hind margin (Fig. 7b).

###### Comparative notes.

See comparative notes of *V.bui* sp. nov.

###### Distribution.

China (Guizhou) (Fig. 11).

##### ﻿ Velia sinensis

Taxon classificationAnimaliaHemipteraVeliidae

Andersen, 1981

B6B5053F-E98B-5EC0-93FF-132027257DB0

Figs 2c, d , 3e, f , 4a–l , 5c , 6e, f , 7c, g , 8g–i , 9e, f

###### Material examined.

2 apterous ♂♂ 2 apterous ♀♀, China, Sichuan Province, Emei Mountain, Jiu Lao Dong Scenic area: 1800–1900 m a.s.l.; 1957-VII-22; Keren Huang leg. (NKUM). 5 apterous ♀♀, China, Sichuan Province, Meishan City, Hongya County, Yanv Lake: 29.6335°N, 103.0858°E; 2013-VII-25; Xin Yu & Xubo Jiang leg. (NKUM). 1 apterous ♂ 7 apterous ♀♀, CHINA, Sichuan Province, Mianyang City, Qianfoshan National Nature Reserve: 29.8880°N, 103.0336°E; 2400 m a.s.l.; 2015-VII-17; Zhen Ye & Chenguang Zheng leg. (NKUM). 1 apterous ♂ 4 apterous ♀♀, China, Sichuan Province, Dujiangyan City, Taian Town, Qingchenghoushan Scenic area: 30.9189°N, 103.4778°E; 918 m a.s.l.; 2014-VII-14; Zhen Ye, Yahui Zhen & Chenguang Zheng leg. (NKUM). 1 apterous ♂ 6 apterous ♀♀, China, Sichuan Province, Ebian County, XinchangTown, Yangziyan Village: 29.2478°N, 103.2631°E; 1022 m a.s.l.; 2017-VII-27; Chenguang Zheng leg. (NKUM).

###### Diagnosis.

**Figure 4.**
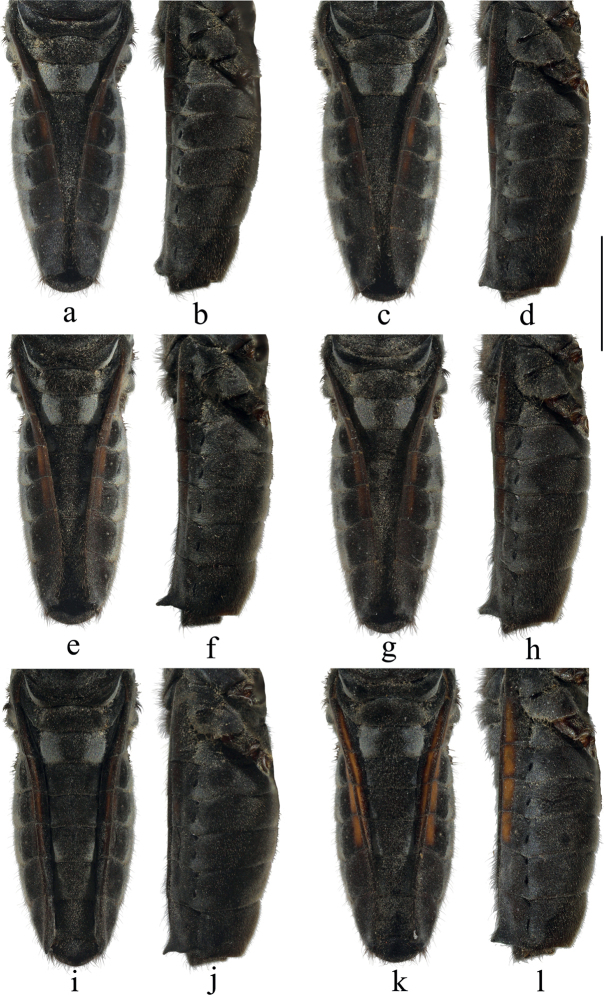
Abdomen of *V.sinensis* (females) from Qianfoshan National Nature Reserve, Mianyang City, Sichuan Province, China **a** connexiva strongly convergent type I, dorsal view **b** connexiva strongly convergent type I, lateral view **c** connexiva strongly convergent type II, dorsal view **d** connexiva strongly convergent type II, lateral view **e** connexiva moderately convergent type I, dorsal view **f** connexiva moderately convergent type I, lateral view **g** connexiva moderately convergent type II, dorsal view **h** connexiva moderately convergent type II, lateral view **i** connexiva slightly convergent type I, dorsal view **j** connexiva slightly convergent type I, lateral view **k** connexiva slightly convergent type II, dorsal view **l** connexiva slightly convergent type II, lateral view. Scale bar: 2.0 mm.

Body large, mainly dark brown to black, commonly dull (Figs 2c, d, 3e, f, 4a–l), but some individuals with conspicuous orange strips along connexiva (Fig. 4k, l); abdominal segment VIII of male small and slightly concaved ventrally (Fig. 6e, f); proctiger of male simple, with rounded hind margin (Fig. 7c).

**Figure 5.**
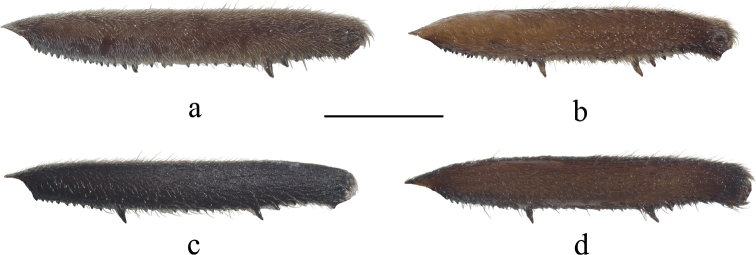
Metafemora of males, showing patterns of spines **a***V.bui* sp. nov. **b***V.longiconnexiva***c***V.sinensis***d***V.tonkina*. Scale bar: 1.0 mm.

**Figure 6.**
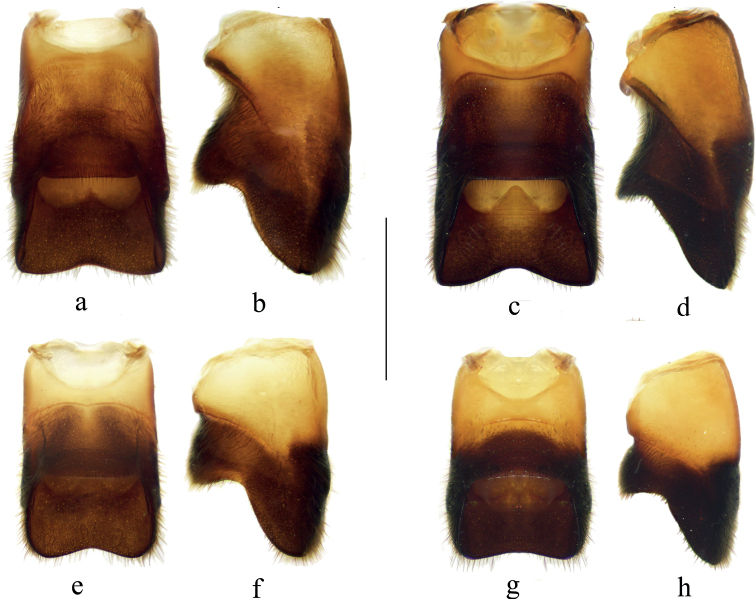
Abdominal segments VIII of males **a***V.bui* sp. nov., ventral view **b***V.bui* sp. nov., lateral view **c***V.longiconnexiva*, ventral view **d***V.longiconnexiva*, lateral view **e***V.sinensis*, ventral view **f***V.sinensis*, lateral view **g***V.tonkina*, ventral view **h***V.tonkina*, lateral view. Scale bar: 1.0 mm.

**Figure 7.**
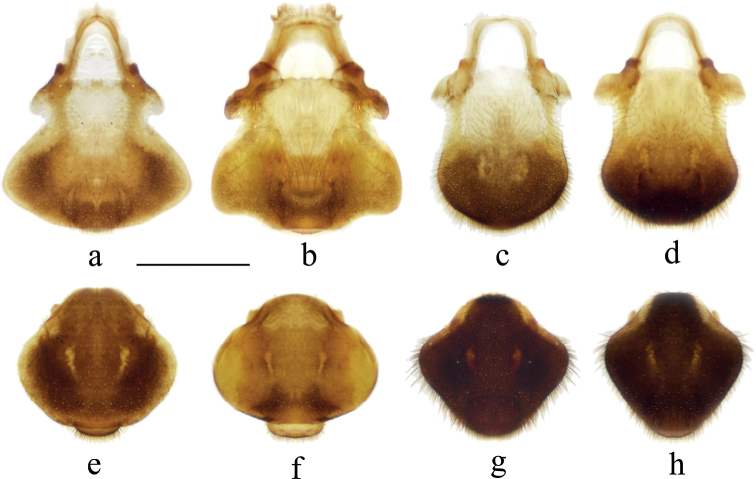
Proctigers of *Velia* spp. **a***V.bui* sp. nov., male **b***V.longiconnexiva*, male **c***V.sinensis*, male **d***V.tonkina*, male **e***V.bui* sp. nov., female **f***V.longiconnexiva*, female **g***V.sinensis*, female **h***V.tonkina*, female. Scale bar: 0.5 mm.

###### Comparative notes.

The comparison between *V.sinensis* and *V.tonkina* has been elucidated by Polhemus and Polhemus (2003) and Tran et al. (2009). In addition, *V.sinensis* can be distinguished from *V.tonkina* by having the basal ends of the lateral sclerites in the endosoma slightly curved laterally (Fig. 9e). In contrast, *V.tonkina* has the basal ends of the lateral sclerites in the endosoma slightly curved inward (Fig. 9g).

###### Habitats.

Some specimens of *V.sinensis* have been observed and collected in the shaded water surface and rock surface near streams (Fig. 10a, c).

###### Distribution.

China (Sichuan) (Fig. 11).

##### ﻿ Velia tonkina

Taxon classificationAnimaliaHemipteraVeliidae

Polhemus & Polhemus, 2003

287AC10B-27E5-5BA1-A266-688ED14FBD65

**Figure 8.**
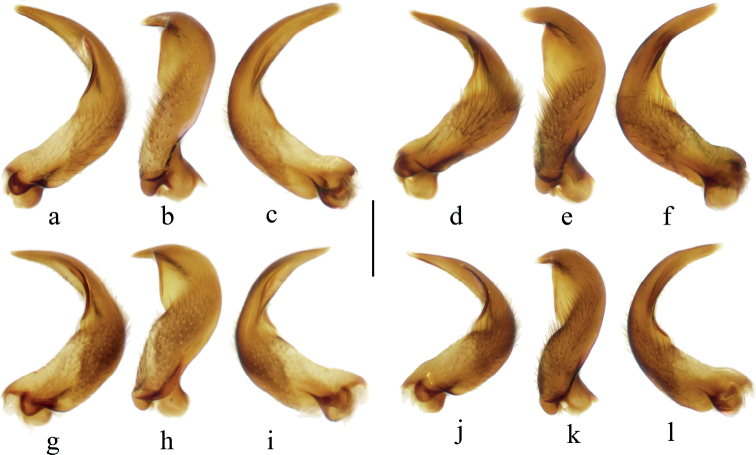
Parameres of males **a***V.bui* sp. nov., external view **b***V.bui* sp. nov., perpendicular view **c***V.bui* sp. nov., internal view **d***V.longiconnexiva*, external view **e***V.longiconnexiva*, perpendicular view **f***V.longiconnexiva*, internal view **g***V.sinensis*, external view **h***V.sinensis*, perpendicular view **i***V.sinensis*, internal view **j***V.tonkina*, external view **k***V.tonkina*, perpendicular view **l***V.tonkina*, internal view. Scale bar: 0.2 mm.

**Figure 9.**
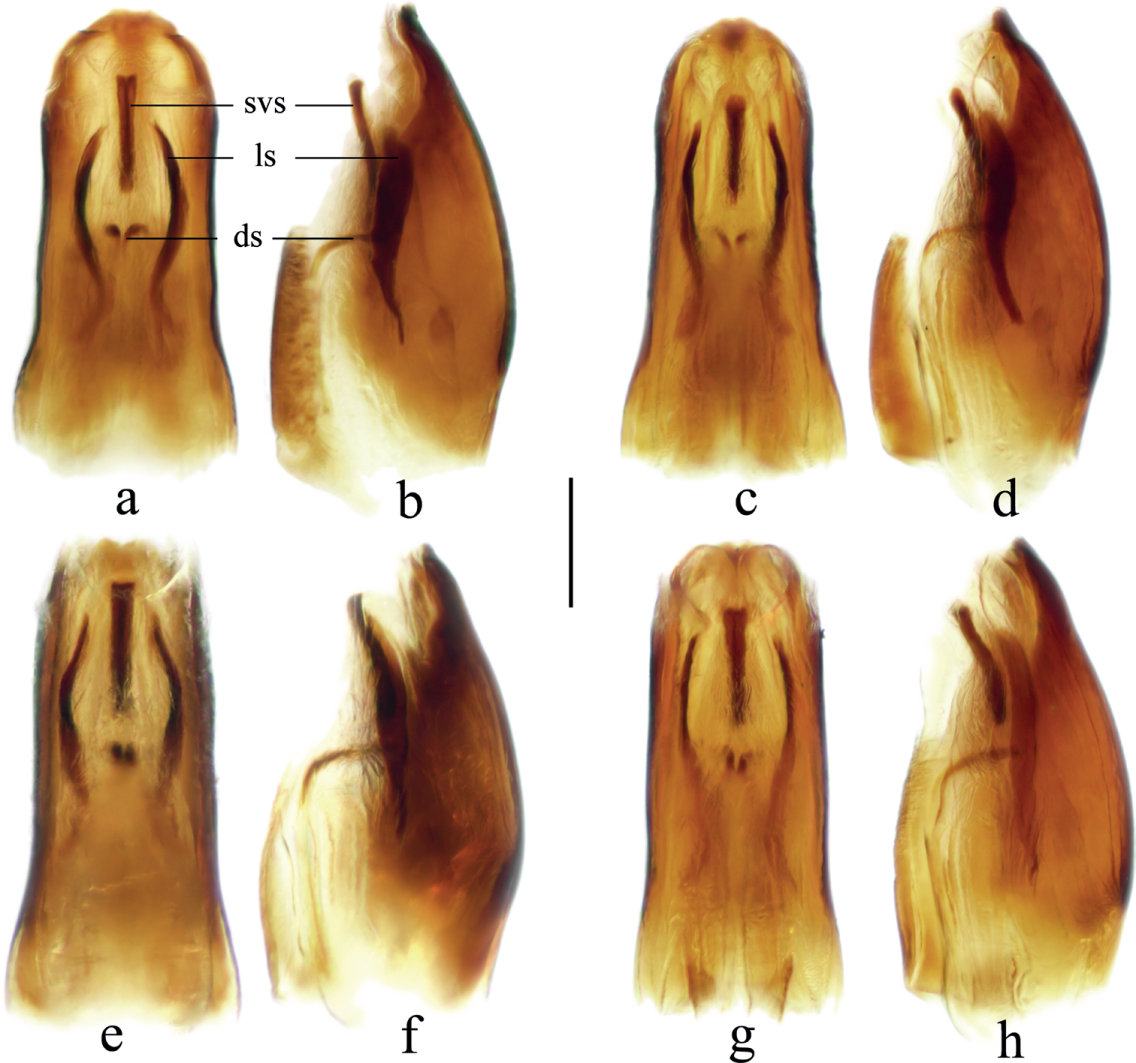
Endosomal structures of *Velia* spp. (males) **a***V.bui* sp. nov., dorsal view **b***V.bui* sp. nov., lateral view **c***V.longiconnexiva*, dorsal view **d***V.longiconnexiva*, lateral view **e***V.sinensis*, dorsal view **f***V.sinensis*, lateral view **g***V.tonkina*, dorsal view **h***V.tonkina*, lateral view. Scale bar: 0.2 mm. (ds = dorsal sclerite, ls = lateral sclerite, svs = secondary ventral sclerite).

Figs 2e, f , 3g, h , 5d , 6g, h , 7h , 8j–l , 9g, h

###### Material examined.

1 apterous ♂ 1 apterous ♀, China, Yunnan Province, Yuxi City, Gasa Town, Shimenxia Scenic area: 23.9688°N, 101.5127°E; 2013m a.s.l; 2016-VIII-01; Zhen Ye leg. (NKUM). 2 apterous ♀♀, China, Yunnan Province, Honghe Pingbian Miao Autonomous County, Dweishan National Reserve: 22.9701°N, 103.7082°E; 2011-IV-16; Zhen Ye leg. (NKUM).

###### Diagnosis.

Body large, mainly dark brown. Connexiva with dark orange strips (Figs 2e, f, 3g, h); connexival spines of female short, dorsocaudally directed, in some specimens strongly reduced (Fig. 3g, h); abdominal segment VIII of male small and ventrally concaved (Fig. 6g, h); proctiger of male simple, with broad rounded hind margin (Fig. 7d)

###### Comparative notes.

See comparative notes of *V.sinensis* and in Tran et al. (2009).

###### Habitats.

**Figure 10.**
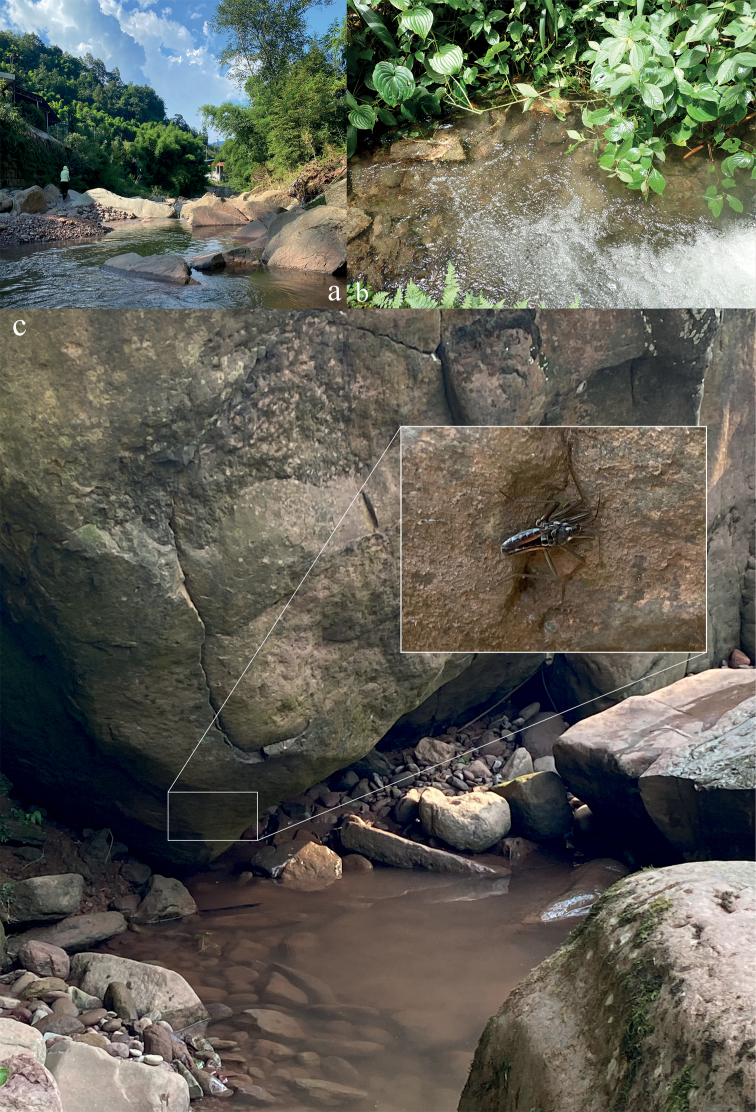
Photographs of habitats of *Velia* spp. **a** habitat of *V.sinensis***b** habitat of *V.tonkina***c** habitat of cryptic area on rock surfaces near the stream of *V.sinensis*.

Some specimens of *V.tonkina* have been observed and collected in the shade of pools surface (Fig. 10b).

###### Distribution.

**Figure 11.**
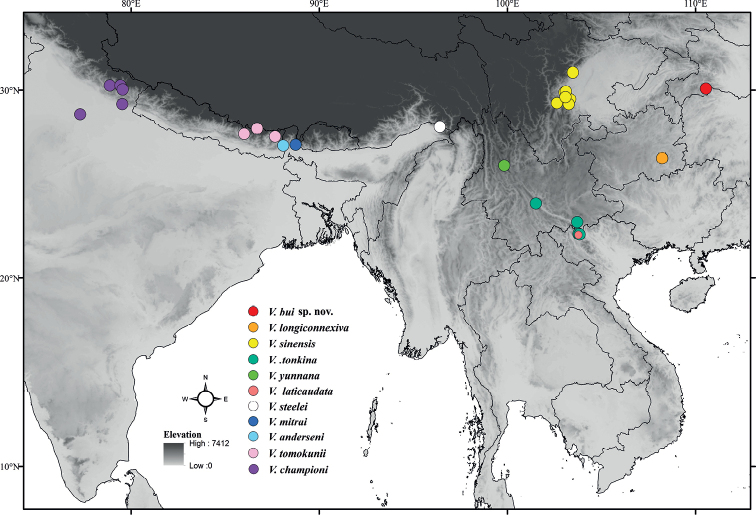
Geographical distribution of subgenus Velia (Cesavelia).

China (Yunnan, first record for China), and Vietnam (Fig. 11).

## ﻿Discussion

Intraspecific variation among female individuals of *V.sinensis* and *V.tonkina* had been noticed and discussed by Tran et al. (2009). In this study, female specimens of *V.sinensis*, collected from one site (i.e., Qianfoshan National Nature Reserve, Mianyang City, Sichuan Prov., China), can be divided into three forms based on the levels of convergence of connexiva: (1) individuals with strongly convergent connexiva (Fig. 4a–d); (2) individuals with moderately convergent connexiva (Fig. 4e–h); and (3) individuals with slightly convergent connexiva (Fig. 4i–l). In addition, the connexival spines and the coloration of stripes along connexiva in the female collected from the same site above are also variable (Fig. 4a–l). Therefore, we speculate that the morphological variation at least within *V.sinensis* might not be attributable to the effects of geographical isolation. This phenomenon needs to be elucidated by subsequent studies based on molecular data.

## Supplementary Material

XML Treatment for Velia (Cesavelia) bui

XML Treatment for Velia longiconnexiva

XML Treatment for Velia sinensis

XML Treatment for Velia tonkina
